# Equity in the use of antithrombotic drugs, beta-blockers and statins among Finnish coronary patients

**DOI:** 10.1186/1475-9276-7-16

**Published:** 2008-06-30

**Authors:** Kristiina Manderbacka, Ilmo Keskimäki, Antti Reunanen, Timo Klaukka

**Affiliations:** 1 National Research and Development Centre for Welfare and Health, Health Services Research, P.O.Box 220, 00531 Helsinki, Finland; 2 National Public Health Institute, Department of health and functional capacity, Mannerheimintie 166, 00300 Helsinki, Finland; 3 Social Insurance Institution, Research Department, P.O.Box 450, 00101 Helsinki, Finland; 4 Tampere School of Public Health, 33014 University of Tampere, Finland

## Abstract

**Background:**

Earlier studies have mainly reported the use of antithrombotic drugs, beta-blockers and statins among hospital patient populations or MI patients. This study aimed to describe the use of these drugs among middle-aged Finnish coronary patients and to identify patient groups in risk of being prescribed inadequate medication for secondary prevention of coronary heart disease.

**Methods:**

One-year follow-up survey data from a random sample of a cohort of coronary patients were used along with register data linked to the survey. The response rate was 54% (n = 2650). The main outcome measures were use of antithrombotic drugs, beta-blockers and statins and the data were analysed using logistic regression analysis.

**Results:**

Among men and women, respectively, 82% and 81% used beta-blockers, 95% and 89% used antithrombotic drugs, and 62% and 59% used statins. Younger men and men from higher socioeconomic groups were more likely to use statins, even after controlling for disease severity and comorbidity. In women, the age trend was reversed and no socioeconomic differences were found. Drug use increased with increased disease severity, but diabetes had only a slight effect.

**Conclusion:**

The use of antithrombotic drugs and beta-blockers among Finnish coronary patients seemed to be rather appropriate and, to some extent, prescription practices of preventive medication varied according to patients' risk of coronary events. However, statin use was remarkably low among men with low socio-economic status, and there is need to improve preventive drug treatment among diabetic coronary patients.

## Background

Numerous randomised trials have shown that drugs for reducing low density lipoprotein (LDL) cholesterol [1], blood pressure [2], and platelet function [3] reduce the incidence of vascular events, such as myocardial infarction (MI), non-fatal stroke and vascular death among high risk individuals, including coronary heart disease (CHD) patients. Combining aspirin or other antithrombotic drugs, beta-blockers and blood cholesterol lowering drugs is thus recommended for secondary prevention of CHD [4]. These recommendations have also been published in Finnish medical journals [5].

In 1995–1996 and in 1999–2000, the EUROASPIRE I and II Group [6] examined the use of aspirin and other antithrombotic drugs, beta-blockers, ACE-inhibitors and lipid-lowering drugs, especially statins, in nine European countries among a population of hospitalised coronary patients aged 70 years or younger. For Finland they reported a prevalence of 82% for antithrombotic drugs, 88% for beta-blockers and 63% for statins in 1999–2000. Strandberg and colleagues [7] reported somewhat lower levels of drug use among home-dwelling elderly (75+ years) coronary patients in Helsinki, Finland in 1998–1999.

Use of services according to need and equal access to health care are fundamental tenets of Finnish health policy [8]. Two dimensions of equity need to be taken into account when evaluating the functioning of the health care system in specific patient groups, such as coronary heart disease patients, namely equal treatment of equals (horizontal equity) and unequal treatment of unequals (vertical equity) [e.g. [9]]. Earlier studies from other countries have reported gender and age differences in the prevalence of use of these drugs among MI patients [10,11] and coronary patients in general [12]. The findings for socioeconomic differences are inconsistent. Some studies have reported socioeconomic disparities in the use of these drugs [11], whereas others have found no differences [12]. Additionally, a recent ecological analysis of GP prescribing practices has reported possible inequities in prescribing rates on the basis of deprivation [13]. In Finland both gender [14] and socioeconomic [15] differences have been reported in the drug treatment of patients after first MI. Male patients and those coming from higher socioeconomic groups were more often prescribed beta-blockers, antithrombotic drugs and cholesterol lowering drugs at discharge from hospital than female patients and those from lower socioeconomic groups. However, Strandberg and colleagues [7] found no socioeconomic differences in drug use among elderly Finnish coronary patients.

Earlier studies have mainly reported the use of these drugs among hospital patient populations or MI patients. The aim of this study was to describe the use of aspirin and other antithrombotic drugs, beta-blockers and statins among middle-aged Finnish coronary patients. So as to evaluate treatment during a stable phase of CHD, we analysed the use of these drugs in a random sample of coronary patients diagnosed from six to seven years prior to our survey. Since the study aimed to identify patient groups in risk of being prescribed inadequate medication for secondary prevention of CHD, it examined the use of the drugs by gender, age, socioeconomic status, disease history and severity, and CHD related comorbidity.

## Methods

### The Finnish drug reimbursement system

Use of medicines in Finnish outpatient care is financed by the patients and the national sickness insurance scheme, run by the Social Insurance Institution (SII). All inhabitants are covered by the scheme, and annually around two-thirds of the population get reimbursement. The share of the scheme of the costs varies depending on the disease. At the time of our survey, if the patient had a severe and chronic illness he/she got a 100% or 75% reimbursement instead of the basic 50%. Coronary heart disease belonged to those diseases which entitled to 75% refund, if the criteria set by the SII were met [16].

### The study sample

A register of patients fulfilling special diagnostic criteria of CHD and thereby eligible for special reimbursement of medicine costs is maintained by the SII. The specific diagnostic criteria for inclusion are (1) chronic angina pectoris symptoms responding to nitrates, and with unequivocal ECG changes (if QS waves are not detected in resting ECG, typical ischaemic changes are required in exercise test), (2) diagnosed MI, (3) a performed revascularisation operation (coronary angioplasty or coronary artery bypass grafting), or (4) CHD diagnosed in angiography. A certificate written by a specialist in cardiology or internal medicine is required to evaluate whether the diagnostic criteria have been met. In January 2001, 5009 Finnish speaking persons aged between 45 and 74 years, and alive at that time and entitled to special reimbursement of medication costs due to CHD in 1994–1995 were drawn for the study sample from the SII register. It was assumed that the treatment situation would be stable among coronary patients some years after the onset of the disease. To guarantee a sufficient number of women, the study sample was first evenly stratified by gender, and random sampling was then applied within each gender group by hospital district to ensure regional representativeness. Structured survey questionnaires asking about sociodemographic background, CHD symptoms, disease history and severity, treatment, comorbidity, and psychosocial factors were sent to the study sample patients in February 2001.

**Figure 1:**
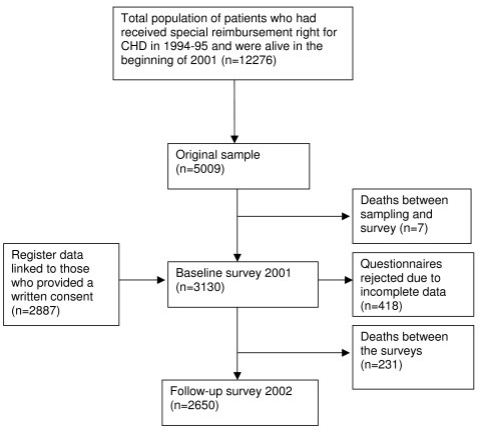
The flow chart of the study sample.

Altogether 3 539 questionnaires were returned; 418 were rejected due to incomplete data (missing values exceeding 20%), leaving 3 121 people in the study sample (62% of the original sample). The baseline survey also asked the participants to provide a written consent for combining their questionnaire data with information about their use of hospital services derived from the Hospital Discharge Register and with sociodemographic information in census data from Statistics Finland. Consent was given by 92% of the respondents. The one-year follow-up questionnaire was sent to respondents of the baseline survey in February 2002. The response rate was 85% (54% of the original sample), resulting in 2 650 respondents (Figure 1).

A separate analysis of non-response showed that women, older persons and those with lower level of education were more likely to be non-responders in the baseline and those with lower level of education in the follow-up. Weights were therefore calculated by gender, age (five-year age groups) and level of education using logistic regression analysis to correct the effect of non-response both between the original sample and baseline respondents and between baseline and follow-up. The study protocol was approved by the Research Ethics Committee of STAKES.

### Variables

The current analyses were based on answers to three questions on drug use in the follow-up survey. Use of beta-blockers, antithrombotic drugs, and statins was analysed separately for each drug group and for all three drug groups combined, based on an open-ended question concerning any prescription drugs used, and its name, dosage and length of use (in days) during the follow-up year. Use of aspirin and other antithrombotic drugs was additionally based on answers to a question about over the counter drugs, plus a separate specific question on use of aspirin or other antithrombotic drugs. Those reporting use of antithrombotic drugs in any of these questions were considered to be users of antithrombotic drugs. The reported trade names were coded according to the Anatomical Therapeutic Chemical (ATC) classification system.

The independent variables were derived from the baseline survey and from register data, which was individually linked to the survey data using the personal identification numbers by the relevant statistical authorities. The identification numbers were removed from the data before handing it to the research team. The *sociodemographic *variables included gender, age, level of education, social class, disposable income and hospital district of residence. *Disease history and severity *were identified by use of short term nitrates at least once a week, occurrence of MI, self-assessed score for exertional chest pain following the New York Heart Association classification, and history of coronary revascularisation. CHD-related *comorbidity *included heart failure and diabetes.

Information about gender and age came from SII and level of education from census data for the year 2000 from Statistics Finland. Education was measured as level of educational achievement, high corresponding to 12 or more years of education, intermediate to 10–11 years, and basic to nine years or less. Data on social class were derived from census data for 2000, and for those outside the labour market from longitudinal census data covering 1970–1995. Family disposable income was derived from census and national tax register data for 2000 and adjusted for family size using the OECD equivalence scale. For statistical analyses income thirds were calculated. Data on hospital district was derived from the 2000 census. Data on hospitalisation due to MI and revascularisation operations in 1990–2001 were derived from the Finnish Hospital Discharge Register for those who consented to the combining of their register and self-reported data (92%). Self-reported information was used for the remainder. The rest of the variables were based on the baseline questionnaire. The chest pain question asked respondents to assess, whether they had exertional chest pain 1 = not at all or only on heavy straining, 2 = walking uphill or ascending stairs, 3 = walking on the level, or 4 = also while resting. A dichotomous variable for exertional chest pain was computed by combining options 2–4.

### Statistical methods

Age standardised proportions were estimated by logistic regression analysis using the predictive margins approach [17]. In this approach, adjusted proportions can be interpreted as the expected response for the individual in group *r *as an average predicted response if everyone in the sample had been in group *r*. Graubard and Korn [17] have generalised the predictive margins approach to non-linear cases, which was used in this study. The total sample was used as the standard population. Logistic regression analysis was also used to analyse the possible effect of the independent variables on drug use, controlling for age only, and age and all other independent variables. Separate models were estimated using the SAS software for the three drug groups and their combination, and men and women were analysed separately. Models were estimated for each of the socioeconomic variables, but since the results were similar with each of them, only the models by income are presented. Since the multivariate analyses produced similar results to the models controlling only for age, only the latter are presented. The results are presented as odds ratios and their 95% confidence intervals.

## Results

There were small variations in the use of beta-blockers, antithrombotic drugs and statins between men and women (Table 1); however, women used antithrombotic drugs significantly less often than men. Half of both men and women reported using all three of these drug categories, and a further 36% of men and 33% of women reported use of two of them, mainly antithrombotic drugs and beta-blockers. If use of only one drug type was reported, antithrombotics were the most common. Table 1 also presents figures for the use of each drug by all independent variables.

**Table 1:** Use of antithrombotic drugs, beta-blockers and statins among men and women.

		**All three drugs**		**Antithrombotics**		**Betablockers**		**Statins**		**n**	
		**Men**	**Women**	**Men**	**Women**	**Men**	**Women**	**Men**	**Women**	**Men**	**Women**
	**Total**	51.6	49.1	93.3	89.1	81.8	80.6	62.1	58.6	1429	1221
**Age (years)**	**45–54**	59.6	45.2	95.0	85.0	86.2	77.4	69.3	52.7	218	93
	**55–64**	54.4	46.0	94.1	87.6	82.5	79.8	65.8	55.0	594	411
	**65–74**	46.2	51.3	91.9	90.5	79.6	81.5	56.1	61.4	617	717
**Income third**	**highest**	55.4	48.0	93.7	89.4	81.8	80.3	68.2	55.1	519	303
	**intermed**	48.6	51.9	94.4	89.0	82.3	81.7	58.7	61.9	456	366
	**lowest**	50.0	50.1	92.0	89.0	81.8	80.9	57.3	60.1	353	468
**MI**	**no**	47.9	47.1	91.7	87.5	79.6	79.2	58.9	56.7	792	916
	**yes**	55.3	57.2	95.5	95.1	85.1	85.1	64.5	66.4	613	276
**Use of**	**less**	51.0	48.4	93.3	89.1	81.9	81.0	61.4	57.8	1211	953
**nitrates**	≥ **1/week**	52.9	53.4	92.6	90.2	82.6	79.4	61.4	62.5	194	246
**Chest pain**	**no**	51.8	46.5	94.1	88.5	80.0	78.3	63.6	57.0	738	391
	**yes**	51.6	50.8	93.1	89.6	84.2	81.7	60.5	60.1	623	758
**Diabetes**	**no**	51.3	48.6	92.9	89.0	81.1	80.8	61.9	58.3	1232	1056
	**yes**	49.9	54.2	95.0	91.0	86.9	81.1	57.7	62.9	197	165
**Heart**	**no**	51.7	49.6	93.4	89.4	82.0	80.7	62.4	59.3	1129	962
**failure**	**yes**	48.9	48.6	92.3	89.1	81.5	81.3	57.3	57.6	300	259
**Revascularisation**	**no**	41.7	40.9	90.5	86.4	80.3	77.9	51.4	51.1	738	891
	**yes**	61.7	72.9	96.0	97.2	83.8	88.4	72.4	80.4	683	322

Age group specific and age-standardised prevalence percentages by income, disease history and severity, and comorbidities.

Among men, age and socioeconomic patterns were found in the use of these drugs (Table 2): younger men and those from higher socioeconomic groups had higher odds for drug use. However, these differences only reached statistical significance for statin use and, for the age pattern, in use of all three drug types combined. Among women the age pattern was reversed, but did not reach statistical significance except for statins. No socioeconomic disparities were found among women. Use of nitrates, diabetes or heart failure did not affect drug use among either gender. Men with exertional chest pain were more likely to use beta-blockers than others, while among women the pattern was similar for each of these drugs, but did not reach statistical significance. In both genders, MI patients and patients who had undergone a revascularisation operation had higher odds for the use of each of these drugs and of all three combined.

**Table 2:** Use of antithrombotic drugs, statins and beta-blockers among Finnish CHD-patients in 2001.

**MEN**		**All three**			**Antithrombotic** **drugs**			**Beta-blockers**			**Statins**		
		**OR**	**95% CI**	**p value**	**OR**	**95% CI**	**p =**	**OR**	**95% CI**	**p value**	**OR**	**95% CI**	**p value**
**Age**	**45–54**	1.00			1.00			1.00			1.00		
	**55–64**	0.80	0.58 – 1.11		0.83	0.40 – 1.72		0.71	0.45 – 1.13		0.87	0.62 – 1.23	
	**65–74**	0.57	0.41 – 0.79	**<0.001**	0.59	0.30 – 1.18	**ns**	0.59	0.37 – 0.93	**ns**	0.57	0.41 – 0.80	**<0.001**
**Income**	**highest**	1.00			1.00			1.00			1.00		
**third**	**intermed**	0.77	0.58 – 1.01		1.16	0.65 – 2.06		1.05	0.74 – 1.50		0.68	0.51 – 0.90	
	**lowest**	0.81	0.60 – 1.08	**ns**	0.73	0.42 – 1.29	**ns**	1.00	0.68 – 1.45	**ns**	0.64	0.47 – 0.87	**<0.01**
**AMI**	**no**	1.00			1.00			1.00			1.00		
	**yes**	1.34	1.08 – 1.67	**<0.01**	1.89	1.17 – 3.04	**<0.01**	1.45	1.09 – 1.95	**<0.05**	1.26	1.01 – 1.58	**<0.05**
**Use of**	**less**	1.00			1.00			1.00			1.00		
**nitrates**	≥ **1/week**	1.09	0.80 – 1.48	**ns**	0.91	0.50 – 1.64	**ns**	1.05	0.70 – 1.57	**ns**	1.00	0.73 – 1.38	**ns**
**Chest pain**	**no**	1.00			1.00			1.00			1.00		
	**yes**	1.00	0.80 – 1.25	**ns**	0.85	0.54 – 1.33	**ns**	1.35	1.01 – 1.80	**<0.05**	0.88	0.70 – 1.11	**ns**
**Diabetes**	**no**	1.00			1.00			1.00			1.00		
	**yes**	0.95	0.69 – 1.29	**ns**	1.48	0.74 – 2.94	**ns**	1.56	1.00 – 2.44	**ns**	0.83	0.61 – 1.14	**ns**
**Heart**	**no**	1.00			1.00			1.00			1.00		
**failure**	**yes**	0.89	0.69 – 1.16	**ns**	0.85	0.52 – 1.39	**ns**	0.97	0.69 – 1.36	**ns**	0.81	0.62 – 1.06	**ns**
**Revasc**.	**no**	1.00			1.00			1.00			1.00		
	**yes**	2.26	1.81 – 2.82	**<0.0001**	2.57	1.60 – 4.13	**0.0001**	1.27	0.96 – 1.68	**ns**	2.48	1.97 – 3.13	**<0.0001**
**WOMEN**		**All three**			**Antithrombotic** ** drugs**			**Beta-blockers**			**Statins**		
		**OR**	**95% CI**	**p value**	**OR**	**95% CI**	**p value**	**OR**	**95% CI**	**p value**	**OR**	**95% CI**	**p value**
**Age**	**45–54**	1.00			1.00			1.00			1.00		
	**55–64**	1.07	0.68 – 1.67		1.17	0.61 – 2.23		1.18	0.69 – 2.02		1.11	0.71 – 1.74	
	**65–74**	1.31	0.86 – 2.01	**ns**	1.58	0.85 – 2.95	**ns**	1.31	0.78 – 2.18	**ns**	1.46	0.95 – 2.23	**<0.01**
**Income**	**highest**	1.00			1.00			1.00			1.00		
**third**	**intermed**	1.14	0.83 – 1.55		0.94	0.58 – 1.54		1.06	0.72 – 1.57		1.29	0.94 – 1.77	
	**lowest**	1.05	0.78 – 1.42	**ns**	0.95	0.59 – 1.54	**ns**	1.01	0.70 – 1.47	**ns**	1.18	0.88 – 1.60	**ns**
**AMI**	**no**	1.00			1.00			1.00			1.00		
	**yes**	1.51	1.16 – 1.97	**<0.01**	2.79	1.60 – 4.89	**<0.001**	1.51	1.06 – 2.15	**<0.05**	1.52	1.16 – 2.00	**<0.01**
**Use of**	**less**	1.00			1.00			1.00			1.00		
**nitrates**	≥ **1/week**	1.21	0.92 – 1.59	**ns**	1.11	0.71 – 1.76	**ns**	0.90	0.64 – 1.27	**ns**	1.20	0.91 – 1.59	**ns**
**Chest pain**	**no**	1.00			1.00			1.00			1.00		
	**yes**	1.19	0.93 – 1.51	**ns**	1.14	0.78 – 1.67	**ns**	1.25	0.93 – 1.68	**ns**	1.13	0.89 – 1.44	**ns**
**Diabetes**	**no**	1.00			1.00			1.00			1.00		
	**yes**	1.27	0.93 – 1.75	**ns**	1.26	0.73 – 2.19	**ns**	1.03	0.69 – 1.54	**ns**	1.23	0.89 – 1.71	**ns**
**Heart**	**no**	1.00			1.00			1.00			1.00		
**failure**	**yes**	0.94	0.72 – 1.23	**ns**	0.96	0.62 – 1.48	**ns**	1.03	0.73 – 1.45	**ns**	0.92	0.70 – 1.20	**ns**
**Revasc**.	**no**	1.00			1.00			1.00			1.00		
	**yes**	3.92	2.99 – 5.15	**<0.0001**	5.55	2.84 – 10.8	**<0.0001**	2.16	1.50 – 3.11	**<0.0001**	3.96	2.95 – 5.33	**<0.0001**

Models for use of each drug controlling only for age, five-year age bands. Odds ratios (OR) and their 95% confidence intervals (CI)

When simultaneously controlling for age, socioeconomic status, disease history and severity, and comorbidity, few changes were detected in the results. The age pattern remained practically unchanged for men, and lost statistical significance among women. Socioeconomic differences remained unchanged in both genders. This was also true for nitrate use and comorbidity among both men and women and for men reporting exertional chest pain. However, among women reporting exertional chest pain, the increased use of beta-blockers (OR 1.45, 95% CI 1.03 to 2.04), statins (OR 1.37, 95% CI 1.03 to 1.83) and all three of these drugs (OR 1.50, 95% CI 1.13 to 1.99) reached statistical significance. Results for history of revascularisation remained similar for both genders and for MI among men; among women MI only reached statistical significance in antithrombotic drug use (OR 2.97, 95% CI 1.53 to 5.80).

When examining more closely the effect of MI and history of revascularisation on drug use, a statistically significant interaction was found among men in the combined use of all three of these drug types. Among MI men, the effect of a history of revascularisation on drug use was smaller (OR 1.64 95% CI 1.16 to 2.32) than among non-MI men (OR 3.03 95% CI 2.24 to 4.09). Among women, revascularisation only had an effect in antithrombotic drug use among non-MI women (OR 9.25, 95% CI 3.36 to 25.5).

## Discussion

The levels of use of antithrombotic drugs, beta-blockers and statins by Finnish patients with established CHD were largely appropriate, but some interesting subgroup differences emerged. The levels of drug use in the present study are similar to earlier findings in Finland [6], although somewhat lower than those reported in EUROASPIRE II and somewhat higher than in the study of home-dwelling elderly in Helsinki. There was also an especially markable difference in the use of statins compared to the study by Strandberg and colleagues [7]. These differences are probably due to differences in the study populations and time periods. Our analyses were based on a randomly sampled cohort of coronary patients whereas EUROASPIRE II analysed hospital patient (MI, coronary revascularisation, or hospital care due to myocardial ischaemia) populations. However, it should be noted that patients in our study had established disease according to at least some objective criteria; those with symptoms but without objective evidence of CHD were not included. The population studied by Strandberg and colleagues [7] was much older than ours. Moreover, coronary patients first qualified for extra reimbursement for statins in 2000, after the Strandberg study.

Drug use decreased with age among men, but the reverse was found among women. Whether these findings indicate less than appropriate health care among older men and excess care among older women is difficult to interpret. The former interpretation may imply the need for more efficient care for elderly men. In line with earlier results [e.g. [12,18]] the likelihood of drug use tended to grow with increased disease severity, although the differences were statistically significant in only a few subgroups. Thus patients with past MI and history of coronary revascularisation, and women with exertional chest pain were more likely to use drugs. Patients with known diabetes also used somewhat more drugs than non-diabetic patients, but the differences were not statistically significant. The fate of coronary patients with diabetes is known to be more serious than that of patients without diabetes [19]. If the present results indicate that Finnish doctors do not consider that diabetes justifies more effective drug treatment of coronary patients, then more physician education is needed.

There were no marked disparities in use of antithrombotic drugs or beta-blockers by socioeconomic indicators. However, after controlling for age, disease history and severity, and comorbity, socioeconomic differences persisted among men in statin use. There could be several reasons for this: whereas aspirin and beta-blockers are relatively inexpensive drugs, statins – even after an increased reimbursement rate – remain more costly for the individual patient, which may explain the lower statin use among less affluent groups. Moreover, the tradition of using antithrombotic drugs and beta-blockers among coronary patients is much longer than for statins. On the other hand, earlier studies both in Finland and elsewhere have reported more effective treatment of CHD among higher socioeconomic groups, including thrombolytic treatment after MI [15] and revascularisation operations after MI [15,20,21] and among coronary patients in general [22,23]. As our analysis was based on questionnaire information, no laboratory examination data were available on possible differences in serum cholesterol levels between socioeconomic groups. However, we were unable to find any Finnish studies reporting higher serum cholesterol levels among more affluent men. Instead, Finnish research has consistently reported higher serum cholesterol levels among lower socioeconomic groups [24].

Our results nevertheless fail to clarify whether socioeconomic differences in statin use are due to physicians' uneven prescription practices or variation in patients' compliance with drug treatment. Several Finnish studies have reported healthier behaviours among higher socioeconomic groups both in terms of dietary fat use and leisure time physical exercise [25].

Earlier studies on hormone replacement therapy and antihypertensive medication have suggested area variation in drug use [26,27]. Area differences especially in secondary prevention practices could have an effect on socioeconomic differences found in our CHD cohort. We carried out additional analysis (random effects model, SAS 8e Mixed procedure) to control the potential effect of regional differences between 20 hospital districts in our results. However, the analysis showed no area effect.

A strength of our study is that it was based on a random sample from a cohort of coronary patients and not merely hospitalised patients. Additionally, the specific criteria used for defining CHD and the approval procedure for special entitlements would have minimised false positive cases. There are some factors, however, that might potentially bias our findings on socioeconomic differences in drug use. First, a possible source of bias is multiple comparisons which could have produced false positive results by chance. Since our results were systematic by all the socioeconomic variables, this is unlikely to be the case. Another issue is selective mortality which is likely to have resulted in fewer respondents from lower socioeconomic groups due to higher mortality before hospitalisation and higher case-fatality from first coronary events [15]. Moreover, in our data those with lower level of education were more likely than others to be non-respondents. To correct the potential bias introduced by selective non-response, weights were calculated to our data. However, weighting assumes that the drug use of respondents and non-respondents is similar within these groups, which may not be the case. Due to selective mortality and the distribution of non-responders, it seems obvious that our results give a conservative estimate of socioeconomic differences rather than an overestimate. This conclusion is supported by an additional analysis of non-response using SII drug register data which revealed that non-respondents were less likely to have had medicine costs for statins (OR = 0.66, 95% CI 0.59 to 0.75) and beta-blockers (OR = 0.82, 95% CI 0.71 to 0.94) reimbursed during 2000 than respondents. No differences were found between respondents and non-respondents in reimbursements for nitrates (OR = 0.92, 95% CI 0.81 to 1.04). This selective non-response means that our estimates for prevalence of beta-blocker and statin use are probably somewhat optimistic. A third potential source of bias is that other than CHD related comorbidity – which is likely to be more prevalent among lower socioeconomic groups – was not controlled for. Since statins are not reported to have major interaction effects with other drugs, the possible mechanism could be either the need to control the total amount of drugs used or high total drug costs. The effect of non-CHD related morbidity on statin use remains an important issue for further analysis. A fourth potential source of bias is recall bias in reporting drug use. We were not able to analyse the impact of recall bias in detail because we did not have register information on medicine use in our data. However, we do not expect there to be systematic socioeconomic bias, since earlier studies have reported high reliability and validity in survey self-reports of medicine use [28].

## Conclusion

According to our results about 50% of Finnish coronary heart disease patients used all three medications, antithrombotic agents, beta-blockers and statins, for preventing new coronary events. We were not able to use clinical information for assessing the appropriateness of the individual patients' treatment but the results seem to indicate a need to evaluate the practices to prescribe statins for coronary heart disease patients. In terms of horizontal equity, socioeconomic differences were observed in statin use possibly indicating more efficient care of more affluent patients or a longer seeding period in the care pattern of less affluent patients with this expensive mode of treatment. In terms of vertical equity more emphasis should evidently be paid to some subgroups, such as diabetic patients in order to improve their care.

## Competing interests

The authors declare that they have no competing interests.

## Authors' contributions

KM participated in the planning of the study questions and analysis, performed the statistical analyses and drafted the manuscript. IK, TK and AR participated in the planning of the study questions and analysis, and were actively involved in the reporting of results and conclusions. All authors read and approved the final manuscript.
